# Prescribing Patterns in Outpatient Clinics of Township Hospitals in China: A Comparative Study before and after the 2009 Health System Reform

**DOI:** 10.3390/ijerph13070679

**Published:** 2016-07-05

**Authors:** Ding Ding, Qingxia Pan, Linghan Shan, Chaojie Liu, Lijun Gao, Yanhua Hao, Jian Song, Ning Ning, Yu Cui, Ye Li, Xinye Qi, Chao Liang, Qunhong Wu, Guoxiang Liu

**Affiliations:** 1 School of Health Management, Harbin Medical University, Harbin 150000, China; dingdingmail0000@126.com (D.D.); 13115305583@163.com (Q.P.); linghanshan@126.com (L.S.); gg73@163.com (L.G.); hyhyjw@126.com (Y.H.); ningninghyd@163.com (N.N.); cuiyu4640@163.com (Y.C.); liye8459@163.com (Y.L.); qixinye19920405@163.com (X.Q.); lchmu2016@163.com (C.L.); 2 School of Psychology and Public Health, La Trobe University, Bundoora 3086, Australia; C.Liu@latrobe.edu.au; 3 The First Affiliated Hospital of Dalian Medical University, Dalian 116000, China; sj19900303@126.com

**Keywords:** antibiotics, prescribing pattern, township hospitals

## Abstract

*Objective:* China introduced a series of health reforms in 2009, including a national essential medicines policy and a medical insurance system for primary care institutions. This study aimed to determine the changing prescribing patterns associated with those reforms in township hospitals. *Methods:* A multi-stage stratified random cluster sampling method was adopted to identify 29 township hospitals from six counties in three provinces. A total of 2899 prescriptions were collected from the participating township hospitals using a systematic random sampling strategy. Seven prescribing indicators were calculated and compared between 2008 and 2013, assessing use of medicines (antibiotics and adrenal corticosteroids) and polypharmacy, administration route of medicines (injections), and affordability of medicines. *Results:* Significant changes in prescribing patterns were found. The average number of medicines and costs per-prescription dropped by about 50%. The percentage of prescriptions requiring antibiotics declined from 54% to 38%. The percentage of prescriptions requiring adrenal corticosteroid declined from 14% to 4%. The percentage of prescriptions requiring injections declined from 54% to 25%. Despite similar changing patterns, significant regional differences were observed. *Conclusions:* Significant changes in prescribing patterns are evident in township hospitals in China. Overprescription of antibiotics, injections and adrenal corticosteroids has been reduced. However, salient regional disparities still exist. Further studies are needed to determine potential shifts in the risk of the inappropriate use of medicines from primary care settings to metropolitan hospitals.

## 1. Introduction

The irrational use of medicines has attracted serious concerns globally. It is costly, not only in terms of human suffering, but also in monetary terms. The irrational use of medicines can lead to exacerbation or prolongation of illness, and cause increased morbidity and mortality, draining scarce medical care resources[1]. The World Health Organization (WHO) estimated that nearly 50% of all medicines in the world are used irrationally. In Europe, at least 25,000 patients die every year because of irrational use of antibiotics, and the costs amounted to more than 1.5 billion EUR annually [2]. Prescribing rates for antibiotics and injections are particularly high in developing countries. In Bangladesh, for example, 78% of prescriptions contained antibiotics and 68.7% included injections [3]. The irrational and over-use of antibiotics, adrenal corticosteroids and injections is common in China. China was viewed as the second largest consumer of antibiotics in the world where it has been estimated that antibiotics account for 30%–50% of consumption of all medicines [4]. Antibiotic prescribing rates in China were too high compared with those in the U.S. and other developed countries, where only 22%–25% of patients were prescribed with antibiotics [5]. What is even more concerning is that 77.07% of prescriptions included injections, as was found in a study in 2005 [6], and high levels of use of adrenal corticosteroids were also found in China (16.8% prescriptions required adrenal corticosteroids in township hospitals in 2001) [7]. This prescribing behavior may bring additional harm to patients in addition to the consumption of the medicines themselves.

The high level of drug usage in China had been driven by several reasons. One is due to a lack of knowledge about the side effects of antibiotics, adrenal corticosteroids and injections abuse in patients [8], there are still many patients who regard antibiotics as “panacea” and believe all disease treatments should include them. Another important reason is that the insufficient government investment and lower level of salaries for doctors lead them to depend on over-prescribing drugs to get extra bonus and pay, so antibiotics, adrenal corticosteroids and injections are more likely to be prescribed in the face of patients’ self-demands. Combined with a lack of strict monitoring on those behaviors, the shortage of qualified medical doctors, along with inappropriate training in primary health facilities jeopardizes the appropriate use of medicines [9]. 

Serious consequences associated with the irrational use of medicines are emerging in China. On average, 14,738,000 incidents of moderate/severe adverse drug reactions of antibiotics are reported every year in China, and approximately 150,000 patients die of these events [5]. The Safe Injection Alliance of China estimated that more than 390,000 people die from unsafe injections every year—51% dying as a result of an adverse drug reaction [10]. Irrational use of antibiotics, injections and corticosteroids will be a public health crisis in the near future unless met with an effective control system. 

In 2009, the Chinese government launched a series of reforms, among which the National Essential Medicines Program (NEMP) was introduced as a key measure to curb the irrational use of medicines and to contain medical expenditures [11]. The supply of medicines in primary care facilities was restricted to those included in the Essential Medicines List (EML). A zero mark-up margin was allowed in relation to prescribing medicines in primary care settings [12]. In addition, Clinical Guideline stipulating clear requirements for antimicrobial use of all hospital levels in China had been established in 2011. Meanwhile, the rapid expansion of the medical insurance system and the increased availability of medicines further improved the consumer accessibility and affordability of medicines. Early evidence shows that the health system reform has been successful in reducing the cost of prescribed medicines, however, over-prescriptions remain at a high level in some regions [13]. This study compared prescribing patterns in a representative sample of township hospitals across three regions (eastern, northern and western) of China between 2008 (pre-reform) and 2013 (post-reform), with an aim to identify changing prescribing patterns in township hospital facilities.

## 2. Methods

A pre-post comparative study design was adopted. The year prior to the introduction of the NEMP (2008) was treated as a pre-reform year. We collected prescription data of the year 2013 and compared them with those in 2008. The study protocol was reviewed and approved by the Institutional Ethical Review Board of Harbin Medical University (Project Identification Code: HMUIRB20160018).

### 2.1. Data Source and Sampling Method

A multi-stage stratified random cluster sampling method was adopted to identify participants. Three provinces, Jiangsu, Heilongjiang and Qinghai, were first identified as representing economically developed (eastern), developing (northern) and under-developed (western) regions of China, respectively. Jiangsu has a population of over 75 million and is highly urbanized; while Heilongjiang and Qinghai has relatively less population density, and more people living in rural areas. Although health expenditure in Jiangsu is higher, it is lower as a percentage of GDP compared with the two less developed provinces (Table 1). 

Two counties (with higher and lower socio-economic development levels) from each province were identified. We randomly selected five township hospitals from each county (except for Qinghai where one township hospital was unavailable). This resulted in a final sample size of 29 township hospitals.

We collected, on average, 100 outpatient prescriptions from each township hospital using a systematic random sampling method. To take into account the seasonal variations in the prevalence of diseases and prescriptions [14], outpatient prescriptions were sampled in March, July and November. All prescriptions dated on the 5th, 15th and 25th of the chosen month were extracted. A total of 2899 prescriptions were included in the data analyses.

### 2.2. Data Analysis

Seven prescribing indicators adapted from the WHO/ International Network for Rational Use of Drugs (INRUD) [15] were used to measure prescribing patterns in township hospitals. The selection of prescribing indicators also considered the NEMP goals in China [12]:

- Percentage of prescriptions requiring antibiotics (PPA)
- Percentage of prescriptions requiring adrenal corticosteroids (PPC)
- Percentage of prescriptions requiring injections (PPI)
- Average number of medicines per prescription (ANMPP)
- Percentage of prescriptions requiring combined use of antibiotics and adrenal corticosteroids (PPA&C)
- Percentage of medicines prescribed from the EML (PEM)
- Average expenditure per prescription (EPP)

PPA and PPC measure the most prevalent problems in the use of a single medicine. The WHO recommended a benchmarking of 20.0%–26.8% for PPA. PPI measures the appropriateness of the administration route of medicines. The WHO recommended a benchmarking of 13.4%–24.1% for PPI. ANMPP and PPA&C measure the degree of polypharmacy. The WHO recommended a benchmarking of 1.6–1.8 for ANMPP. WHO’s benchmarking for PPC and PPA&C is not available [16]. The combined use of antibiotics and adrenal corticosteroids has attracted particular concerns in China [17]. PEM and EPP measure financial affordability of prescribed medicines. Medicines listed in the EML are usually cheaper than those not listed. Theoretically, EML may also fit better with the competency of primary care workers who have limited medical training, reducing irrational prescribing [18]. We compared the prescribing indicators between the year of 2008 (pre-reform) and 2013 (post-reform) by region. Normality of distribution was examined by QQ plots and Shapiro-Wilk test, Kruskal-Wallis H-test was applied to analyze the non-normal distribution data. Changes in categorical variables were tested by chi-square tests. Statistical significance was accepted when *p* values were equal to or less than 0.05. Data analyses were performed using SPSS Statistics Version 17.0 (IBM, Chicago, IL, USA).

## 3. Results

### 3.1. Characteristics of Prescription Recipients and Township Hospitals

In 2008, more than half (55.7%) of the prescription recipients were male patients and 50.4% were male patients in 2013 (χ^2^ = 8.041, *p* = 0.005). The prescription recipients had a mean age of 42.93 ± 23.37 years in 2008 and 44.98 ± 22.35 years in 2013, respectively. There was a significant difference in the age structure of prescription recipients (χ^2^ = 17.245, *p* = 0.008). In township hospitals, the number of practicing physicians showed an increasing trend between 2008 and 2013 (27.01% in Heilongjiang and 15.7% in Jiangsu), except in Qinghai province (only increased by 0.59%). In Jiangsu province, the average number of beds was approximately four times higher than that of other two provinces in 2013. Compared with 2008, the share of government subsidy to hospitals revenue all increased, with Qinghai enjoying the highest increased (19.4%), followed by Heilongjiang (15.9%), while the proportion of medical income to total hospital revenue didn’t increase much, with a slight fall in Qinghai (Table 2).

### 3.2. Changing Patterns of Prescriptions

Statistically significant changes in all seven indicators were found between 2008 and 2013 (Table 3).

The percentage of medicines prescribed from the EML increased from 50% in 2008 to 85% in 2013. The average number of medicines and costs per-prescription dropped by about 50%. On average, a nine to thirty percentage point drop in PPA, PPI, PPC and PPA&C was observed.

### 3.3. Regional Differences in Changing Prescribing Patterns

Similar changing prescribing patterns were found across the three provinces (Table 4). Overall, Jiangsu demonstrated a lower degree of changes, despite its relatively good performance in PPA, PPI, ANMPP and PEM. Jiangsu had the highest level of use of adrenal corticosteroids.

Qinghai experienced greater drops in PPA (by 49.5%), PPI (by 64.0%) and ANMPP (54.7%), compared with 16.2% drop of PPA in Heilongjiang (*p* = 0.000), 44.0% drop of PPI in Jiangsu (*p* = 0.000) and 45.7% drop of ANMPP in Jiangsu (*p* = 0.000). Its average number of medicines per prescription remained the largest, still higher than what the WHO recommended.

Although Heilongjiang performed the worst in PPA, PPI and PEM, its changes in PPC (by 72.3%) and PEM (by 134.6%) were the most dramatic compared to the other two provinces.

### 3.4. Antibiotic Prescriptions

A significant decline in antibiotic prescriptions was found in all three provinces, albeit in various degrees (Table 5). Qinghai had the largest drop in antibiotic prescriptions, with PPA reducing from 68.5% in 2008 to 34.6% in 2013. Heilongjiang had the smallest drop in prescriptions for antibiotics, with only a 13.1% percentage point reduction in the combined use of two or more antibiotics and no significant change in prescribing rates of single antibiotics. 

## 4. Discussion

Improving the rational use of medicines is considered a priority for the health system reform agenda in China. This is particularly important for township hospitals which serve as a central hub of China’s primary health care system. Unfortunately, the inappropriate and overuse of medicines in township hospitals was prevalent. A study of 221 township hospitals in 2001 found that, on average, one prescription contained four types of medicines [7], more than double the number recommended by the WHO. Our study shows that such a high level of use of medicines did not decline much prior to the 2009 health reform. 

This study revealed that significant changes in prescribing patterns occurred in township hospitals since the introduction of the NEMP in 2009. With prescribed medicines coming predominantly from the EML, the number of drugs per prescription was reduced, and so did the average expenditure per prescription. The average number of medicines per prescription (1.76) in 2013 has met the standards (1.6–1.8) recommended by the WHO, lower than that (2.2) in Brazil [19].

Prescribing rates for antibiotics, adrenal corticosteroids and injections in township hospitals all dropped significantly in 2013 compared with those in 2008. On average, the percentage of prescriptions including antibiotics dropped from more than 55% in 2008 to 38.4% in 2013, signifying a great achievement. The prescribing rates for antibiotics in many developing nations exceeds 60%, such as in Pakistan, Indonesia and Mozambique (60%–70%) and Hai Phong (67%) in Vietnam [1,20,21]. The current level of antibiotic use in township hospitals in China remains in the middle range of Asian and African countries (27%–63%) [1], but it is still high and exceeds the level (20.0%–26.8%) recommended by the WHO. In developed nations, prescribing rates for antibiotics were generally low, ranging from approximately 15% for adults in the USA (1995–2002) to around 30% in Spain and France [22]. 

It seems that prescribing rates for antibiotics had been decreasing continuously since 2009. Song and colleagues [23] noticed a further drop in the percentage of prescriptions requiring antibiotics in 2013 (38.4%) compared to 39.97% in 2010 and 41.72% in 2009. They also found that the percentage of prescriptions containing two or more antibiotics in Chinese township hospitals is low (3.8%) compared with their own past data for 2010 (18.5%) and Robert’s findings in Nigeria (16.8%) [20,24].

A similar declining trend in the use of adrenal corticosteroids is also evident. Despite significant variances in PPC (ranging from 5.5% to 18.5%) prior to the 2009 reform, reductions of PPC were achieved in all three provinces. The overall use of adrenal corticosteroids (3.7%) in 2013 was lower than that found in urban community health services in China (7.8% in 2007; 7.4% in 2008; 7.5% in 2009) [17]. The percentage of the combined use of antibiotics and adrenal corticosteroids in 2013 also dropped to a level (2.3%) much lower than those revealed in previous studies [25].

The percentage of prescriptions requiring injections dropped to 24.5% in 2013, still slightly higher than the level the WHO recommended level (13.4%–24.1%), but it is low compared with Uganda (48%) and Mongolia (48%) [26,27].

Several policy initiatives may have contributed to China’s success in changing prescribing patterns in primary care settings. First, the enforcement of the NEMP and the zero mark-up policy associated with prescribed medicines works in favor of the rational use of medicines [13]. As shown in Table 3, the coverage of NEMP reached from 49.6% in 2008 to 85% in 2013, which means that majority areas has exposed to NEMP impact, as one of the policy requirements, only essential medicines could be prescribed in primary facilities and the exclusion of certain corticosteroids, antibiotics and injections from the EML seem to have reduced the prescription of such drugs. Besides, zero mark-up requires that primary facilities couldn’t profit from drug sales and ensure the essential medicines are available at procurement cost [28]. Meanwhile, to further consolidate its policy effect, China also implemented performance-based salary system in primary health institutions to replace the previous revenue-based bonus system, this changing prescribing incentives for doctors also been found in Xin Zhang’s study [29]. The limited selection of medicines included in the EML fits better with the competency of primary care workers and made them losing financial interest in over-prescription. 

Second, the rapid expansion of medical insurance schemes also played a significant role. By the end of 2012, 98.26% of the rural population had been covered by it. By setting up differential polices for reimbursing essential and non-essential medicines (100% for essential drugs and much lower rate for non-essential drugs), it further added up the effect of NEMP on prescribing behavior. Besides, various payment reform of NCMS have also been piloted to different degree, such as the payment method of global budget, together with setting up deductible and ceiling for reimbursement, the intensity of over-prescription reduced, Wang’s study also proved it [30].

As one of the key indicators of national health reform, the appropriateness of drug prescription has been monitored at township hospitals. With all above measures combined, which led to the effect of enhanced cost awareness of doctors and patients, resulting in reductions in the unnecessary use of medicines [31]. Finally, China published its clinical guidelines on antibiotic prescriptions in 2004, which was followed by regulations in prescription management (in 2006) and prescribing behavioral assessment (in 2010). Prescribing indicators have been incorporated into the performance assessments of medical doctors and medical institutions [4].

It is interesting to note that less developed regions may have benefited more from the reforms than their wealthier counterparts. Qinghai, an underdeveloped province, experienced the most dramatic changes. Despite a higher number of medicines per prescription, it now has the lowest prescribing rates for antibiotics, adrenal corticosteroids and injections, as well as the lowest expenditure per prescription. The possible explanation may lie in an intensified central government budget transfer to poor areas of China together with required counterpart funding from local governments (especially in the poverty-stricken western area) to support the implementation of NEMP in primary health facilities. According to WHO, the increasing funding of government plays a very important role on promoting rational drug use [32]. This study shown that the poorest Qinhai province enjoyed the highest increase in government investment (19.4%), followed by Heilongjiang (15.9%), this dramatic change also prompted more appropriate drug use in primary health facilities.

Evidence shows that actions targeting both providers and consumers are equally important in promoting the rational use of medicines [8]. Health care managers need to pay more attention to the training and supervision of medical workers, as well as the appropriate arrangement of workloads and performance management. Meanwhile, consumers need to be empowered to take increasing responsibility for safety of health care. This can be done through the increased transparency of health care information and the improved health literacy of consumers [33].

## 5. Conclusions

In conclusion, significant changes in prescribing patterns are evident in township hospitals in China. Over-prescriptions of antibiotics, injections and adrenal corticosteroids have reduced. However, salient regional disparities still exist. Further studies are needed to determine potential shifts in the risk of the irrational use of medicines from primary care settings to metropolitan hospitals. The results of this study should be interpreted in light of several limitations. First, we only selected two years in the pre-post reform comparisons. Second, only three provinces were selected in this study, which may limit its representativeness. Third, we are not able to conduct risk-adjustments of the prescribing indicators due to the unavailability of data. 

## Figures and Tables

**Table 1:** Populations, health spending and health workforce in Jiangsu, Heilongjiang and Qinghai.

Characteristics	Jiangsu		Heilongjiang		Qinghai	
	2008	2013	2008	2013	2008	2013
Population (million)	76.77	79.39	38.25	38.35	5.54	5.78
Rural population (million)	35.09	28.49	17.06	16.34	3.27	2.98
Population density (people/km^2^)	342	277.68	36.07	34.55	4.53	4.13
GDP (billion)	3098.2	5975.3	831.4	1445.5	101.9	212.2
Health expenditure as a percentage of GDP (%)	3.16	3.74	5.62	6.73	5.87	7.73
Number of township hospitals	1396	1064	919	996	404	405
Annual outpatient visits to township hospitals (million)	67.1	77.9	9.25	9.51	2.40	2.58
Number of physicians per thousand population in rural areas	0.69	1.28	0.89	1.18	0.86	1.26

Data source: China health statistical yearbooks.

**Table 2:** Characteristics of township hospitals in sample areas.

Characteristics	Heilongjiang		Jiangsu		Qinghai	
	2008	2013	2008	2013	2008	2013
Proportion of practicing physician to the general medical staff (%)	25.62	32.54	34.07	39.42	25.22	25.37
Average number of beds	10.5	14.3	49.7	51	10.3	12.7
Proportion of government subsidies to total hospital revenue (%)	45.37	52.58	40.25	46.05	54.89	65.56
Proportion of medical income to total hospital revenue (%)	5.02	5.37	3.45	3.79	3.23	3.02

**Table 3:** Changing prescribing patterns in township hospitals from 2008 to 2013.

Prescribing Indicator	2008	2013	Difference (95% CI)	χ^2^/*h*	*p*
PPA (%)	58.1	38.4	−19.7 (−23, −16)	113.53	0.000
PPC (%)	12.7	3.7	−9.1 (−11, −7.1)	102.39	0.000
PPI (%)	54.1	24.5	−29.6 (−33, −26)	266.97	0.000
ANMPP	3.41	1.76	−1.65 (−1.16, −2.14)	580.36	0.000
PPA&C (%)	17.4	6.1	−11.3 (−14.6, −8.1)	223.43	0.000
PEM (%)	49.6	85.0	35.4 (32, 39)	416.55	0.000
EPP	65.18	30.97	−34.21 (−6.05, −62.37)	137.63	0.000

Notes: Percentage of prescriptions requiring antibiotics (PPA); Percentage of prescriptions requiring adrenal corticosteroid (PPC); Percentage of prescriptions requiring injections (PPI); Average number of medicines per prescription (ANMPP); Percentage of prescriptions requiring combined use of antibiotics and adrenal corticosteroid (PPA&C) Percentage of medicines prescribed from EML (PEM); Average expenditure per prescription (EPP).

**Table 4:** Comparison of changing prescribing patterns across the three provinces (2008 and 2013).

Prescription Indicator	Jiangsu			Heilongjiang			Qinghai		
	2008	2013	Trend	2008	2013	Trend	2008	2013	Trend
PPA (%)	59.2	36.7	↓ *	54.3	45.5	↓ *	68.5	34.6	↓ *
PPC (%)	18.5	6.2	↓ *	8.3	2.3	↓ *	5.5	1.8	↓ *
PPI (%)	43.2	24.2	↓ *	66.9	30.6	↓ *	55.5	20.0	↓ *
ANMPP	3.22	1.75	↓ *	3.11	1.22	↓ *	4.86	2.20	↓ *
PPA&C (%)	15.3	3.7	↓ *	10.5	2.0	↓ *	2.6	1.0	↓ *
PEM (%)	63.5	90.2	↑ *	34.1	80.0	↑ *	45.5	83.0	↑ *
EPP	65.62	41.56	↓ *	64.11	26.78	↓ *	35.21	21.64	↓ *

Notes: ↑ increasing trend; ↓ decreasing trend; * *p* < 0.05 compared between 2008 and 2013; Percentage of prescriptions requiring antibiotics (PPA); Percentage of prescriptions requiring adrenal corticosteroid (PPC); Percentage of prescriptions requiring injections (PPI); Average number of medicines per prescription (ANMPP); Percentage of prescriptions requiring combined use of antibiotics and adrenal corticosteroid (PPA&C) Percentage of medicines prescribed from EML (PEM); Average expenditure per prescription (EPP).

**Table 5:** Antibiotic prescriptions in township outpatient clinics.

Province	Prescriptions Requiring Antibiotics			Prescriptions Containing only One Antibiotic			Prescriptions Containing Two or More Antibiotics		
	2008 (*n* (%))	2013 (*n* (%))	Difference in Percentage (95% CI)	2008 (*n* (%))	2013 (*n* (%))	Difference in Percentage (95% CI)	2008 (*n* (%))	2013 (*n* (%))	Difference in Percentage (95% CI)
Jiangsu	394 (58.2)	220 (36.7) *	−21.5 (−28, −18)	286 (43.3)	192 (32.1) *	−11.2 (−17, −6)	98 (14.8)	28 (4.3) *	−10.5 (−13, −7)
Heilongjiang	293 (54.3)	182 (45.5) *	−8.8 (−15, −2)	199 (36.9)	165 (41.3)	4.4 (−2, 11)	94 (17.4)	17 (4.3) *	−13.1 (−17, −9)
Qinghai	137 (68.5)	173 (34.6) *	−33.9 (−42, −26)	99 (49.5)	158 (31.6) *	−17.9 (−26, −10)	38 (19.0)	15 (2.8) *	−16.2 (−22, −10)
Total	814 (58.1)	675 (38.4) *	−19.7 (−17, −10)	584 (41.7)	515 (34.4) *	−7.3 (−11, −4)	230 (16.4)	60 (3.8) *	−12.6 (−15, −10)
χ^2^	12.162	12.289		10.949	11.522		2.54	2.067	
*p*	0.002	0.002		0.004	0.003		0.281	0.356	

Notes: CI = confidence interval; * *p* < 0.05 in changes from 2008 to 2013.
